# Supplementary material for: Citizens’ Juries: When Older Adults Deliberate on the Benefits and Risks of Smart Health and Smart Homes
Source: Healthcare (Basel). 2019 Apr 1;7(2):54. doi: 10.3390/healthcare7020054 (PMC6627999; doi:10.3390/healthcare7020054)
Supplement: Supplementary file 1 [file healthcare-07-00054-s001.pdf]

Citizen’s Jury Pre-session Survey

1. How often do you use technology such as the following; mobile phone, motion sensors or alert systems?

a) Several times a day
b) Sometimes
c) Rarely
d) Never

2. How does “Smart City Nottingham” make you feel?

a) Interested about future opportunities
b) Not interested
c) Concerned about technology

3. Do you think that it’s important for older people to use new technologies?

a) Yes, really important
b) Quite important
c) Not really important
d) Don’t know

4. Would you like to be able to influence the design of such technologies?

a) I do not see the point, nobody will listen to me
b) Yes, but I do not know how
c) Definitely, we should be able to shape or future of assistive technologies

5. Who should design and implement the health applications for well-being?

a) Technology developers
b) Technology consumers
c) Local government
d) A representative mix of all the above

Please Turn Over

6. Do you think that Smart Cities have…

a) too much influence over the future healthcare for older adults
b) the right amount of influence over the future healthcare for older adults
c) a little influence over the future healthcare for older adults
d) no influence at all over the future healthcare for older adults

7. Have you ever think about the ethical consequences of health technologies?

a) Yes – often
b) Yes – sometimes
c) Yes - A little
d) No

8. Do you think you can influence the way the health technology industry is advancing?

a) Yes, a lot
b) Yes, sometimes
c) Yes, a little
d) No

9. Do you think that you should have more influence in how Smart Cities are advancing?

a) Yes
b) No
c) Maybe
d) Don't know

Citizens' Jury Post-session Survey

1. Who should be accountable if smart technologies go wrong?

a) The manufacturer
b) The commissioners
c) The health services
d) Other
e) Don't know

2. Did you learn anything new today about assistive technologies for health promotion and self-care?

a) Yes, a lot
b) Yes, a little
c) No
d) Don't know

3. Did you come up with any new ideas today about how we could make Smart Cities more accessible to older adults?

a) Yes, lots of them
b) Yes, a few
c) No
d) Don't know

Please Turn Over

4. On a scale of 1 to 10 (with 1 meaning very little and 10 meaning very much), how much do you agree with the following statements:

1) Smart Cities promote inequalities among citizens that do not have access to technology
2) Replacing human with technology is a good idea
3) The benefits of health technology for society exceed the risks
4) Smart City initiatives have the potential to reach to more people
5) I want to know more about the impact that Smart Cities can have on people
6) I will try to use health technologies more often
7) Smart Cities bring more opportunities to its older citizens
8) The ethical consequences of health technologies are minimal
9) Smart Cities should not influence the future healthcare for older adults
10) I would like to be have a say on the way Smart Cities are advancing

5. On a scale of 1 to 10 (with 1 meaning very little and 10 meaning very much), how much do the following statements apply to you:

1) I'm confident that I can influence societal opinion regarding the implementation of health technologies within Smart Cities
2) Nobody is going to listen to what older adults say about Smart Cities
3) I've changed my mind today about smart solutions for improving health and well-being of older people
4) When I think about the social implications that can be expected from health technologies, I feel more confident about my own opinion
5) I would like to influence decision making and relevant policy regarding the implementation of health technologies among older adults
